# Production of functional recombinant antibodies in *Dictyostelium discoideum*

**DOI:** 10.1186/s13104-025-07314-z

**Published:** 2025-06-04

**Authors:** Cyril Guilhen, Tania Jauslin

**Affiliations:** Biomedical Sciences, Faculty of Medicine, University of Geneva, Geneva, Switzerland

**Keywords:** *Dictyostelium discoideum*, Recombinant antibody production, Immunofluorescence

## Abstract

**Objective:**

Recombinant antibodies are essential reagents for diagnostics, research, and therapy. Numerous production methods have been developed, each of them with its strengths and weaknesses. In this study we evaluated the ability of *Dictyostelium discoideum* cells to produce and secrete functional antibodies.

**Results:**

Three recombinant antibodies targeting tubulin, CISD1 or CD8β proteins, respectively, were successfully produced and secreted by *D. discoideum* cells. Electrophoretic analysis of these antibodies revealed a degradation product, resulting from proteolytic cleavage at the linker peptide connecting the scFv portion to the Fc fragment. Removal of this linker suppressed the proteolytic cleavage. Finally, immunofluorescence analysis confirmed that all three antibodies recognized their target antigen in a specific manner. This study represents the first demonstration that functional recombinant antibodies can be produced in *D. discoideum* cells.

**Supplementary Information:**

The online version contains supplementary material available at 10.1186/s13104-025-07314-z.

## Introduction

Recombinant antibodies play a pivotal role in diagnostics, therapeutics, and research due to their unparalleled specificity and affinity towards target antigens [1, 2]. Since the 1990s, numerous cell types have been employed for their production, each with its own set of advantages and disadvantages. Mammalian cell lines, like Chinese hamster ovary (CHO) cells, are widely used to produce therapeutic antibodies [3]. Their ability to produce antibodies with human-like post-translational modifications is crucial to reduce the risk of immunogenicity associated with non-human glycosylation patterns. However, mammalian cells are difficult and expensive to grow, notably due to the high cost of culture media and of growth factors and the need for a CO_2_-enriched atmosphere. These considerations have fuelled the search for alternative expression systems, particularly to produce antibodies used for diagnostic and research applications [4].

Alternative solutions for recombinant antibody production include other eukaryotic organisms like yeasts, or prokaryotes such as bacteria. These organisms are cost effective and grow faster than mammalian cells. However, they have their own limitations. Recombinant proteins produced in the bacterial cytosol remain unglycosylated and frequently aggregate into non-functional forms [5]. Yeast cells exhibit a mammalian-like secretory apparatus. However, in yeast, the sugars attached to asparagine residues are typically large and formed by the addition of multiple mannose residues, whereas mammalian glycans are smaller and incorporate other sugars like galactose and sialic acid [6, 7].

In this study, we tested the ability of *Dictyostelium discoideum* cells to produce and secrete functional antibodies. *D. discoideum* has been largely used as a model system to study cell motility, chemotaxis, phagocytosis, and multicellular development. *D. discoideum* stands out as an interesting candidate for recombinant antibody production for various reasons. First, it grows in a simple and cheap medium in the absence of exogenous growth factors. Second it grows at room temperature (ideally 20–26 °C) without the need for a CO_2_ enriched atmosphere. Third, it divides rapidly, with a division time of approximately 12 h. Fourth, it can easily be grown as a concentrated cell suspension. Fifth, *D. discoideum* can easily be genetically manipulated, and numerous molecular tools and protocols are available to achieve stable or transient transfection [8]. Sixth, the secretory pathway of *D. discoideum* resembles that of yeast or mammalian cells and it achieves both N- and O-linked glycosylation devoid of the large polymannose glycans [9]. However, there is a dearth of literature on recombinant antibody production in these cells. In this study, we evaluated *D. discoideum*'s capacity to produce and secrete three functional recombinant antibodies.

## Main text

### Methods

#### Production of recombinant antibodies in *D. discoideum* cells

Recombinant antibodies were produced as mini-antibodies with the antigen-binding single-chain fragment variable (scFv) fused to a Fc fragment (Supplementary Fig. 1). The coding sequences of recombinant antibodies were synthesized (GeneArt, Invitrogen) after optimizing codons to maximize production in *D. discoideum* (Table 1). The scFv is a fusion protein made up by the variable domains of the heavy (VH) and light (VL) immunoglobulin chains linked by a short peptide linker (L1: GGGGSGGGGSGGS). The scFv was linked to the Fc by a second peptide linker (L2: RSPSGPISTINPCPPCKECHKCP). The Fc portion comprises the last two domains (CH2 and CH3) of mouse IgG2B (Uniprot: P01867). To facilitate cloning of scFv in prepSC3, two restriction sites (*Kpn*I and *BamH*I) were placed between the signal sequence and the linker L2 connecting the scFv to the Fc (Supplementary Fig. 1a). The signal peptide encoding sequence (Signal P) of CfaD (Uniprot Q54TR1) was placed upstream of the transgene.

**Table 1:** Antibodies produced in this study (ABCD: AntiBodies Chemically Defined Database; https://web.expasy.org/abcd/)

Recombinant antibody	Accession number in ABCD database	References
anti-alpha tubulin	ABCD_AA345	[14]
anti-CISD1	ABCD_RB251	[15]
anti-CD8β	ABCD_AJ517	[16]

The prepSC3 plasmid contains an Actin 15 promoter which drives the expression of the recombinant antibody transgene, a *D. discoideum* polyadenylation and termination signal (pA) [10, 11] (Supplementary Fig. 1), a beta-lactamase gene (*bla*), a bacterial origin of replication (*ori),* a *D. discoideum* Ddp2 *ori* [12] and a neomycin selection marker (*neo*) under the control of the Actin 6 promoter for the selection of transfected amoeba with geneticin. The recombinant vector was transfected by electroporation into *D. discoideum* DH1-10 strain [13], referred to in this study as non-transfected (NT). Cells were grown at 21 °C in HL5 medium supplemented with tetracyclin (16 µg/mL). 24 h after electroporation, geneticin was added to a final concentration of 10 μg/mL and transfected cells were cloned by limiting dilution in 96-well plates. Individual clones were screened by dot blot assay analysis to check for expression of recombinant antibodies. Production of recombinant antibodies was then achieved by cultivating cells under agitation (180 rpm) for five days in a 250 mL Erlenmeyer flask containing 60 mL of cell culture. At a final cell density of approximatively 10^7^ cells/mL, cells were pelleted (320 g; 4 °C; 5 min) and supernatants were harvested and centrifuged (2900 g; 20 min; 4 °C) to remove cellular debris.

#### Evaluation of recombinant antibody concentrations by dot blot assay

*D. discoideum* cell culture supernatants (5 µL) were deposited and dried on a nitrocellulose membrane. The membrane was blocked for 2 h in PBS containing 0.1% (v/v) Tween 20 and 7% (w/v) milk, then incubated with horseradish peroxidase (HRP) coupled goat anti-mouse IgG (Biorad #170-6516, dilution 1:3,000) and finally washed five times for 5 min in PBS-Tween 0.1% (v/v). The signal was revealed by enhanced chemiluminescence (ECL) (Millipore) using a PXi-4 gel imaging systems (Syngene). Signal intensities of various dilutions of supernatant (dilutions performed in HL5 medium) were compared to signals obtained from a control recombinant antibody (ABCD_RB602) with a known concentration.

#### Biochemical characterization of recombinant antibodies

*D. discoideum* cell culture supernatants (15 mL) were mixed with 40 µL of resin coupled with either protein G (Cytiva #17061801) or protein A (Thermo Scientific #20333) pre-washed 3 times with 1 mL of PBS. After 1 h of incubation on a wheel at 4 °C, the resin was recovered and washed four times with 1 mL of PBS. Antibodies attached to the resin were eluted with 60 µL of reducing or non-reducing sample buffer [20.6% (w/v) sucrose, 100 mM Tris pH6.8, 10 mM EDTA, 0.1% (w/v) bromophenol blue, 4% (w/v) SDS, ± 6% (v/v) β-mercaptoethanol]. 20 µL of each sample were separated by electrophoresis on an acrylamide gel 4–20% (SurePAGE Bis–Tris, Genscript #M00655) (200 V, 30 min). Acrylamide gels were washed in distilled water and revealed using Page Blue colorant (Thermo Fisher Scientific #24620). After a 24 h incubation period at 4 °C, colorant was removed, and gels were finally washed in distilled water.

#### Functional characterization of recombinant antibodies by immunofluorescence

To test anti-CISD1 and anti-CD8β antibodies, HEK293 cells (grown in DMEM, Gibco #11960044, supplemented with 10% FBS) were transiently transfected 2 days before the experiment with a vector coding for either the human CISD1 protein (Uniprot Q9NZ45) or the mouse CD8α (Uniprot #P01731) and CD8β proteins (Uniprot #P10300) [17]. Non-transfected (NT) or transfected HEK293 cells were fixed with methanol at -20 °C for 2 min then washed once in PBS and once in PBS + 0.2% (w/v) BSA (PBS-BSA) during 10 min. Cells were then incubated for 20 min with either 1 µg/mL of antibody produced in *D. discoideum* (*D. discoideum* supernatants diluted in PBS-BSA) or in HEK293 cells as described previously [15, 18, 19]. The cell culture supernatant of NT *D. discoideum* cells was used as a negative control. After 3 washes (10 min) with PBS-BSA, cells were incubated for 30 min in PBS-BSA with secondary goat anti-mouse IgG conjugated to AlexaFluor-488 (1:400, Molecular Probes, #A21235). After 3 washes (10 min) with PBS-BSA, cells were mounted on slides (Menzel-Gläser, 76 × 26 mm) with Möwiol (Hoechst) + 2.5% (w/v) DABCO (Fluka 33480). Pictures were taken using a Zeiss LSM800 confocal microscope, with a 63 × Neofluar oil immersion objective.

#### Mass spectrometry

Bands of interest were excised from the Page Blue colorant stained SDS-PAGE gel and stored in 10% (v/v) ethanol solution until mass spectrometry analysis. Samples were analyzed by LC-ESI-MS/MS as described previously [20]. The mass spectrometry proteomics data have been deposited to the ProteomeXchange Consortium via the PRIDE [21] partner repository with the dataset identifier PXD053343.

## Results

To test the ability of *D. discoideum* cells to produce functional antibodies, we used transfected cells expressing the sequence from three different recombinant antibodies obtained from the Antibodies Chemically Defined Database (ABCD; https://web.expasy.org/abcd/). These antibodies target tubulin (AA345), the mitochondrial protein CISD1 (RB251), or the T-cell surface CD8β glycoprotein (AJ517). To direct these recombinant antibodies into the secretory pathway, we placed the sequence encoding the signal peptide of the *D. discoideum* CfaD protein upstream of the transgenes (Supplementary Fig. 1). CfaD protein is abundantly found in the supernatant of amoebic cultures under conditions similar to those in our study (suspension culture with agitation) [22, 23].

**Fig. 1:**
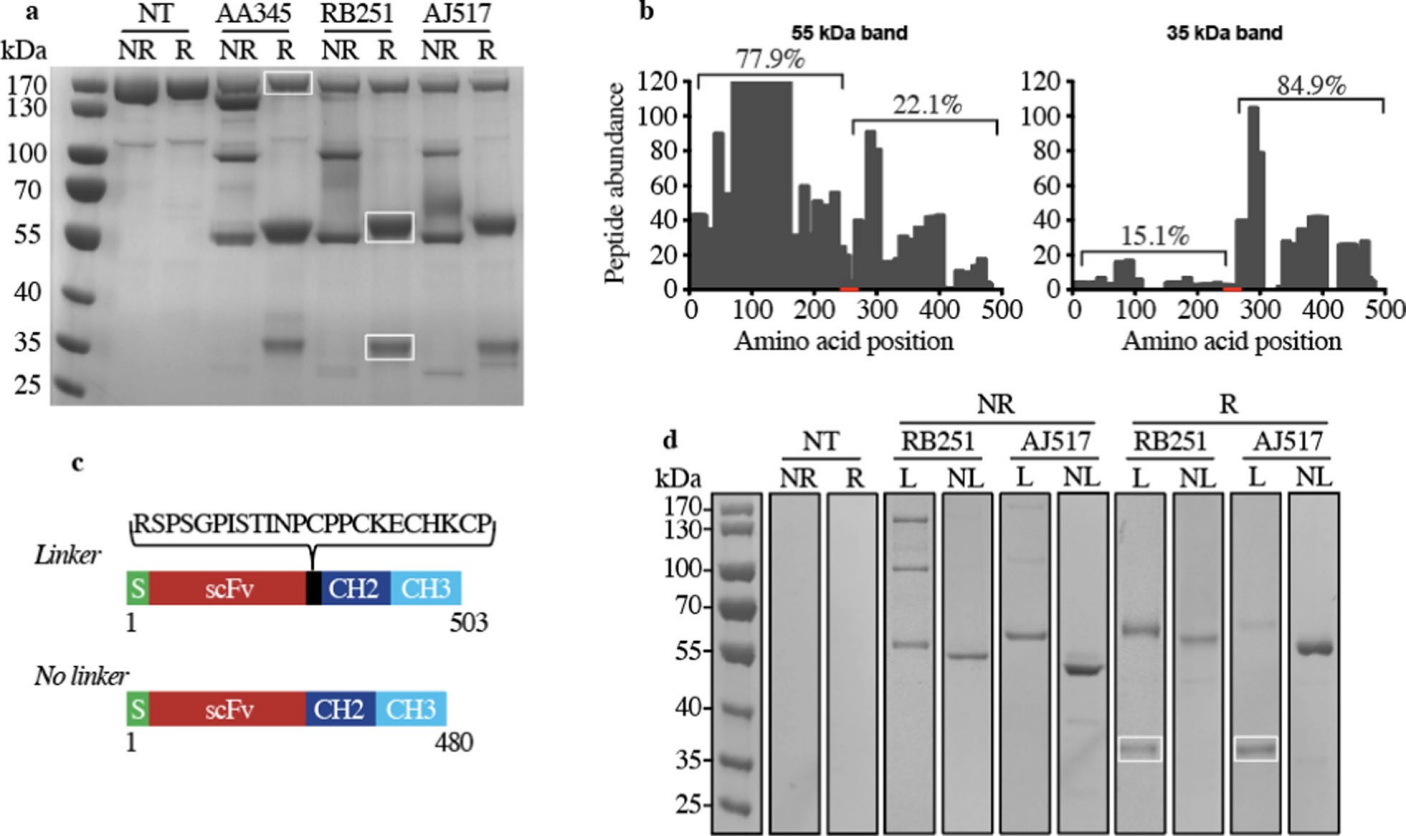
Characterization of recombinant antibodies by SDS-PAGE gel electrophoresis. **a** Protein composition of purified antibodies obtained from either non-transfected *D. discoideum* cells (NT) or AA345, RB251 or AJ517 transfected cells. Proteins attached to the protein G resin were eluted with either non-reducing (NR) or reducing (R) sample buffer. Molecular mass markers are indicated on the left. The three bands framed in white were cut and analyzed by mass spectrometry. **b** Characterization by mass spectrometry of the 55 kDa and 35 kDa bands observed in panel A. These bands were analyzed by mass spectrometry and the obtained peptides were mapped against the 503 amino acids of the RB251 recombinant antibody. Histogram represents the number of peptides mapping with the antibody RB251 in function of the amino acid position. The red line represents the amino acid positions of the linker peptide in the RB251 construct connecting the antigen-binding scFv to the mouse IgG2B Fc. The percentage of peptides mapping either upstream or downstream from the linker is also indicated. **c** Upper part: schematic representation of the linker and no linker versions of RB251 protein sequence with the signal peptide (S) and the single-chain variable fragment (scFv) linked to the CH2 and CH3 domains of mouse IgG2B. Lower part: the DNA sequence encoding for the peptide linker “RSPSGPISTINPCPPCKECHKCP” was removed from the DNA construct and the resulting expression vector (called RB251-NL) was transfected *in D. discoideum* cells. **d** Protein composition of purified antibodies obtained from either non-transfected *D. discoideum* cells (NT), RB251 or AJ517 transfected cells with (L) or without (NL) the linker. Proteins attached to the protein A resin were eluted with either non-reducing (NR) or reducing (R) sample buffer. Molecular mass markers are indicated on the left. The two bands framed in white correspond to the 35 kDa degradation product

We then used protein G coupled to a sepharose resin to purify the antibodies present in the cell culture supernatants. For all three antibodies analyzed, migration under reducing conditions (R) revealed a major band around 55 kDa, the expected size of the monomeric scFv-Fc fragment (Fig. 1a). In non-reducing condition (NR) two bands were visible at 55 and 100 kDa, which likely correspond to the monomeric (theoretical size: 52 kDa) and dimeric versions of the antibody, respectively (Fig. 1a). A non-specific band around 170 kDa was present in all conditions. Mass spectrometry analysis revealed that it is an unrelated protein (RliB; UniProt #Q54YG8) (Supplementary Table 1). The use of protein A instead of protein G for antibody purification eliminated this nonspecific band (Fig. 1d and Supplementary Fig. 2).

In reducing conditions for all three antibodies, the presence of a weak band around 35 kDa suggested that a small proportion of the recombinant antibodies produced was truncated (Fig. 1a). To confirm this interpretation and to locate the cleavage site, the 35 kDa and 55 kDa bands (framed in white) were analyzed by mass spectrometry, and the obtained peptides were aligned against the 503 amino acids of the RB251 antibody. 84.9% of the peptides sequenced from the 35 kDa band aligned downstream of the L2 linker connecting the scFv to the Fc fragment (Fig. 1b). A similar distribution pattern was observed with the 35 kDa bands from the AA345 and AJ517 conditions (data not shown). The same analysis of the full-length scFv-Fc fragment from the 55 kDa band showed a different distribution, with most of the peptides (77.9%) aligning upstream of the L2 linker (Fig. 1b). A similar distribution pattern was also observed with the 55 kDa bands from the AA345 and AJ517 conditions (data not shown). These results suggest the existence of a proteolytic site in the L2 linker connecting the scFv to the Fc fragment (Supplementary Fig. 1). To test this hypothesis, the linker peptide (23 amino acids) was removed to create "no-linker" (NL) RB251 and AJ517 antibodies (Fig. 1c). Despite several attempts, we were unable to obtain clones expressing the AA345 antibody without the L2 linker.

Electrophoretic mobility of antibodies (Fig. 1d and Supplementary Fig. 2) revealed that removing the L2 linker prevented the partial degradation of antibodies, as evidenced by the disappearance of the 35 kDa band (framed in white in Fig. 1d) under reducing conditions. As expected, the bands around 55 kDa, corresponding to a scFv-Fc monomer, were slightly smaller in the NL version than in the full-length version (L) (Fig. 1d). The removal of the L2 linker also resulted in the elimination of interchain disulfide bonds for both constructs since the 100 kDa band, corresponding to antibody dimers, disappeared when the linker was removed (Fig. 1d). The presence of four cysteines, essential for the formation of disulfide bonds in the L2 linker sequence, supports this interpretation (Fig. 1c).

**Fig. 2:**
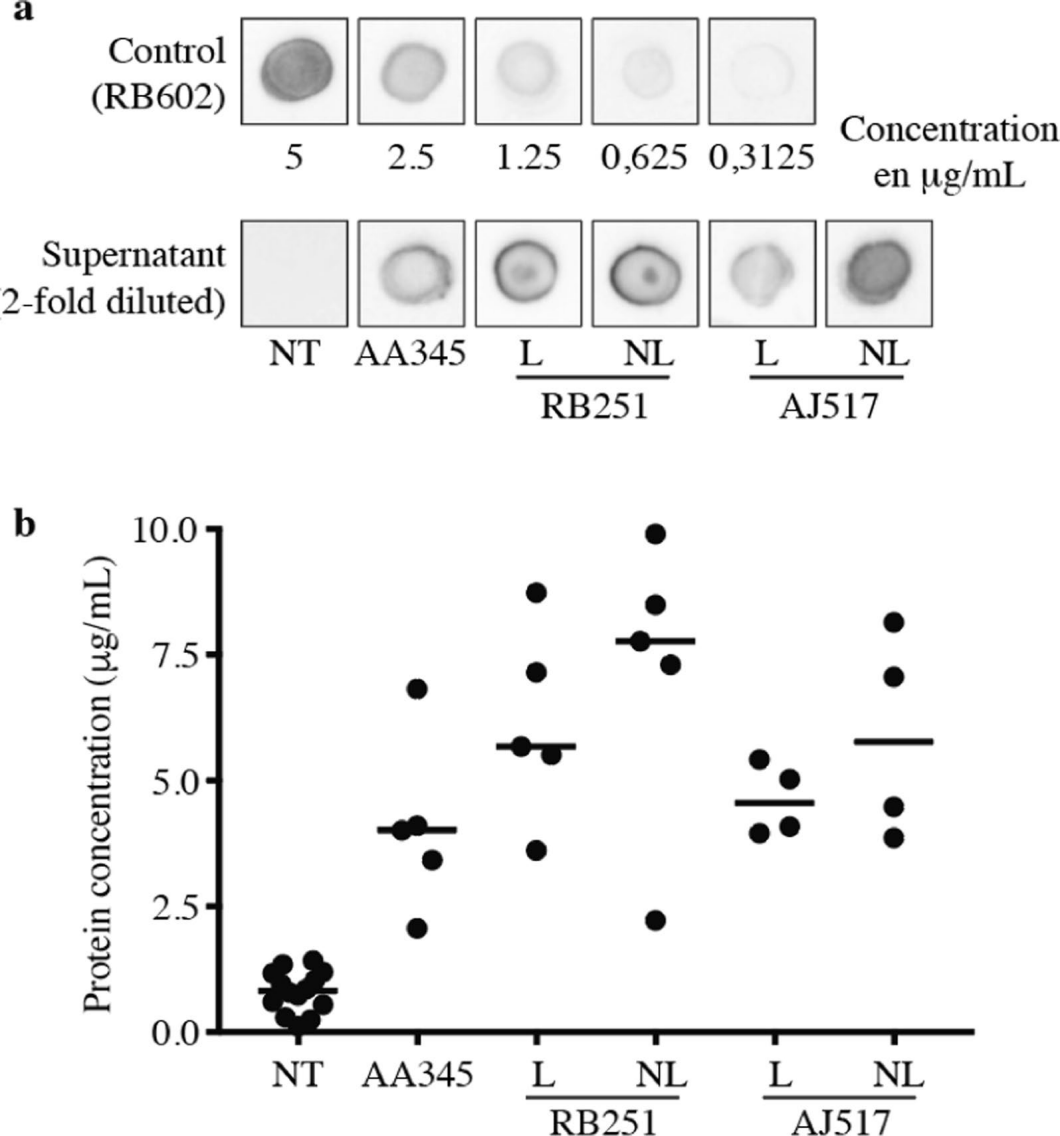
Quantification of recombinant antibodies in supernatant by dot blot assay. **a** Signal intensities of twofold diluted supernatants were compared to signals obtained from a control recombinant antibody (ABCD_RB602) with a known concentration. **b** Comparison with signals obtained from the RB602 control antibody was used to determine concentrations in µg/mL of each of the obtained supernatant. N = 5 independent experiments (4 independent experiments for AJ517 transfected cells). NT: non-transfected cells; L – NL: AA345, RB251 or AJ517 transfected cells with (L) or without (NL) the linker

The concentration of recombinant antibodies in the *D. discoideum* cell culture supernatants was estimated by dot blot assay. After 5 days of culture under agitation at a final cell density of approximatively 10^7^ cells per mL of medium, 5 µL of cell supernatant was deposited on a nitrocellulose membrane. Recombinant antibodies present on the membrane were revealed with an HRP-coupled anti mouse antibody. The intensity of the dots obtained with each recombinant antibody was compared to the dots obtained with a reference antibody of known concentration (RB602) (Fig. 2a). AA345, RB251, and AJ517 antibodies (with the linker) were produced at concentrations of 4 µg/mL, 5.7 µg/mL and 4.6 µg/mL, respectively. In the absence of linker, RB251 and AJ517 antibodies were detected at 7.8 µg/mL and 5.8 µg/mL, respectively, in the supernatant of transfected cells (Fig. 2b). Altogether, the results indicate that (i) the three recombinant antibodies are produced at similar concentrations in *D. discoideum* cells, and (ii) the removal of the linker does not affect the production yield.

**Fig. 3:**
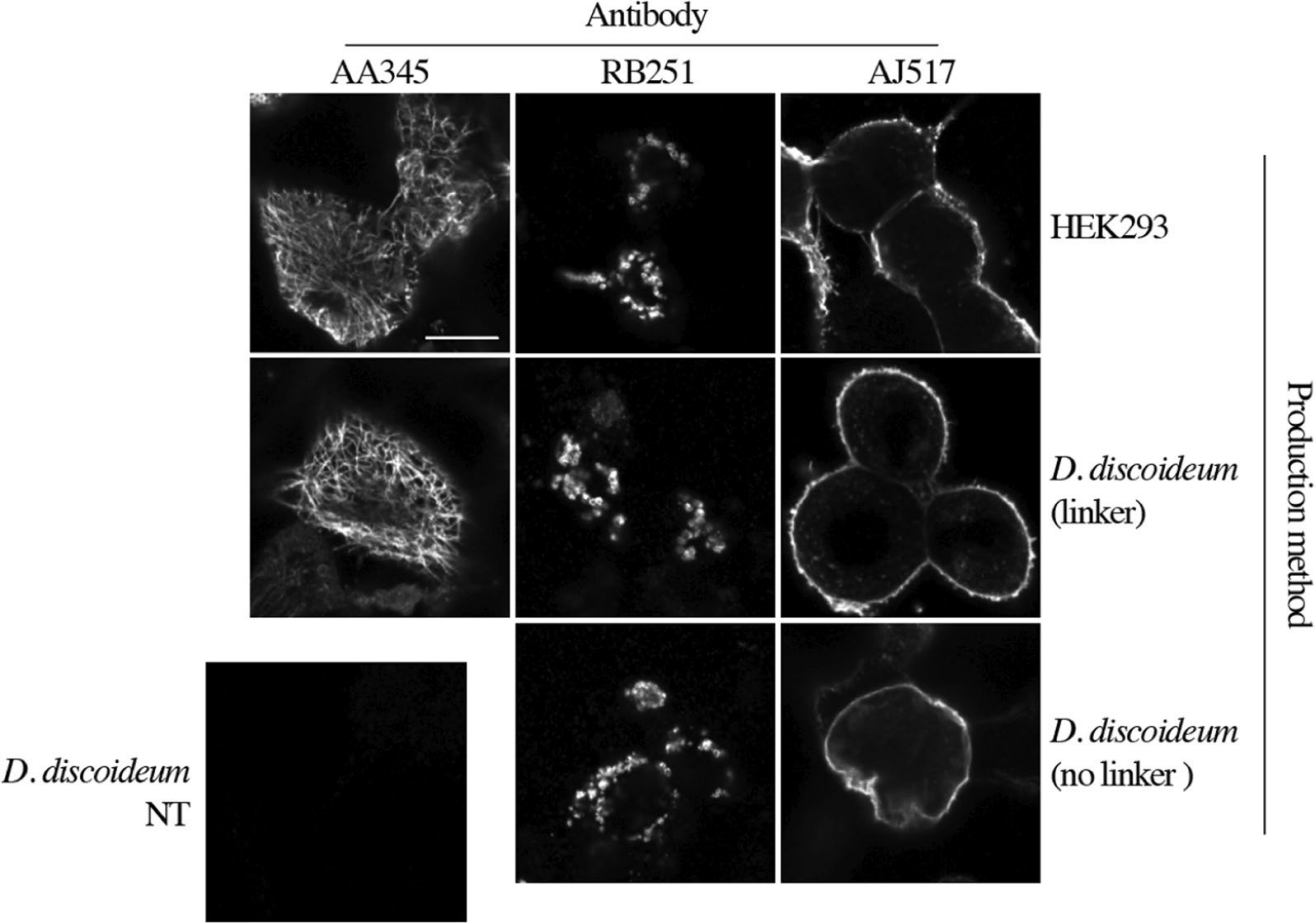
Functional characterization of recombinant antibodies by immunofluorescence. Recombinant antibodies with or without linker secreted in supernatants of *D. discoideum* cells were used to label HEK293 cells. As a positive control, recombinant antibodies produced in mammalian HEK293 cells at 1 µg/mL were used (upper part). As a negative control, supernatant of non-transfected (NT) *D. discoideum* cells was used (lower left part). AA345, RB251 and AJ517 correspond to anti-tubulin, anti-CISD1 and anti-CD8β antibodies respectively. Scale bar: 10 µm

Finally, the ability of recombinant antibodies produced in *D. discoideum* to recognize their target was tested by immunofluorescence. AA345 successfully labelled a network of cytoskeletal filaments typical of the microtubule cytoskeleton in mammalian cells (Fig. 3). Immunolabeling with the RB251 revealed mitochondrial clusters, which are typically observed in cells overexpressing CISD1 [15]. AJ517 recognized the cell surface CD8β protein in transfected HEK293 cells (Fig. 3) [19]. As a control, equivalent signals were obtained with the same recombinant antibodies produced in HEK293 cells and used at the same concentration (Fig. 3). Supernatant from NT amoeba did not label any cellular structure. Importantly, the removal of the linker did not affect the ability of RB251 and AJ517 to recognize their target (Fig. 3).

## Discussion

In this study, we developed a system for producing bivalent recombinant antibodies in an scFv-Fc format using the amoeba *D. discoideum*. Our results demonstrate the successful production of three functional recombinant antibodies targeting tubulin, CISD1 and CD8β as evidenced by their specific binding in immunofluorescence assays. The successful assembly of scFv-Fc fusion proteins into bivalent antibody-like molecules suggests that *D. discoideum* supports the correct folding and functional assembly of complex immunoglobulin-derived constructs. For all three antibodies, *D. discoideum* cell culture media contained approximately 5 µg/mL of antibodies. Higher production yields can be reached in other optimized production systems (e.g., 120 µg/mL with HEK293 cells [24], or 10 µg/mL in the yeast *Saccharomyces cerevisiae* [25] or the bacteria *E. coli* [26]). Several features of the expression vector could be optimized to increase recombinant antibody production in the amoeba: the promoter sequence, the Kozak-like sequence or the signal sequence. Increasing plasmid copy number per cell is another potential strategy to boost transcript levels. For this purpose, the prepSC3 plasmid could be transfected into *D. discoideum* cells containing the REP open reading frame [27], allowing extra-chromosomic plasmid replication via the Ddp2 replication origin (Supplementary Fig. 1) [12]. Additionally, optimizing culture conditions such as incubation time and agitation speed could further improve yields.

Like natural IgG antibodies, antibodies produced in the scFv-Fc format dimerize to form a bivalent structure stabilized by disulfide bonds in the hinge region [28]. In our construct, the L2 linker was removed, eliminating these disulfide bonds. While this does not affect antigen recognition, as shown in immunofluorescence experiments (Fig. 3), it may weaken dimer stability. The original linker could be modified to remove the proteolytic site while retaining the cysteines essential for disulfide bond formation.

This system represents a promising alternative to more traditional eukaryotic expression models, particularly for research applications where small- to medium-scale production is sufficient and ease of genetic manipulation is advantageous. The inherent benefits of *D. discoideum*, such as its capacity for post-translational modifications and low culture costs, make it a compelling candidate for further development in recombinant antibody production. In this context, particular attention should be given to glycosylation, as it plays a critical role in the stability, immunogenicity, and biological activity of recombinant antibodies [29]. Further studies will be necessary to characterize the post-translational modifications, especially glycosylation patterns, of antibodies produced in *D. discoideum*, and to assess their suitability for more advanced biomedical applications.

## Limitations

- Only the scFv-Fc minibody format was tested in this study, which restricts the conclusions regarding the amoeba’s ability to express other antibody formats, such as full-length IgG, Fab fragments, or nanobodies.
- The antibody production yield in *D. discoideum* (~ 5–8 µg/mL) is low compared to other expression systems (e.g., HEK293: 120 µg/mL; *S. cerevisiae*: 10 µg/mL).
- Optimization of the L2 linker is needed to reduce susceptibility to proteolytic cleavage.
- Glycosylation remains to be characterized to assess its impact on antibody function.
- The study did not include a detailed cost–benefit analysis of *D. discoideum* for recombinant antibody production compared to established expression systems.

## Supplementary Information

Supplementary Material 1. **Supplementary Fig. 1:** Design of the expression vector prepSC3-scFv to produce recombinant antibodies in D. discoideum cells. **a** Schematic overview of the prepSC3 plasmid with the scFv fragment cloned in KpnI/BamHI. **b** Schematic of a minibody composed of a dimer of scFv-Fc fragments. (Signal P: signal peptide encoding sequence; VH and VL: variable domains of the heavy (VH) and light (VL) immunoglobulin chains; scFv: single-chain fragment variable; L1 and L2: peptide linkers; pA: D. discoideum polyadenylation and termination signal; neo: neomycin selection marker; ori: E. coli origin of replication; bla: beta-lactamase encoding gene.

Supplementary Material 2. **Supplementary Fig. 2:** Full-length SDS-PAGE gels electrophoresis used to create the panel d in Figure 1. Each lane (numbered from 1 to 10) in panel d was obtained from 4 different gels (a, b, c and d).

## Data Availability

The mass spectrometry proteomics data have been deposited to the ProteomeXchange Consortium via the PRIDE [21] partner repository with the dataset identifier PXD053343.
