# Supplementary figures and images for: Production of functional recombinant antibodies in Dictyostelium discoideum
Source: BMC Res Notes. 2025 Jun 4;18:246. doi: 10.1186/s13104-025-07314-z (PMC12139333; doi:10.1186/s13104-025-07314-z)

**a**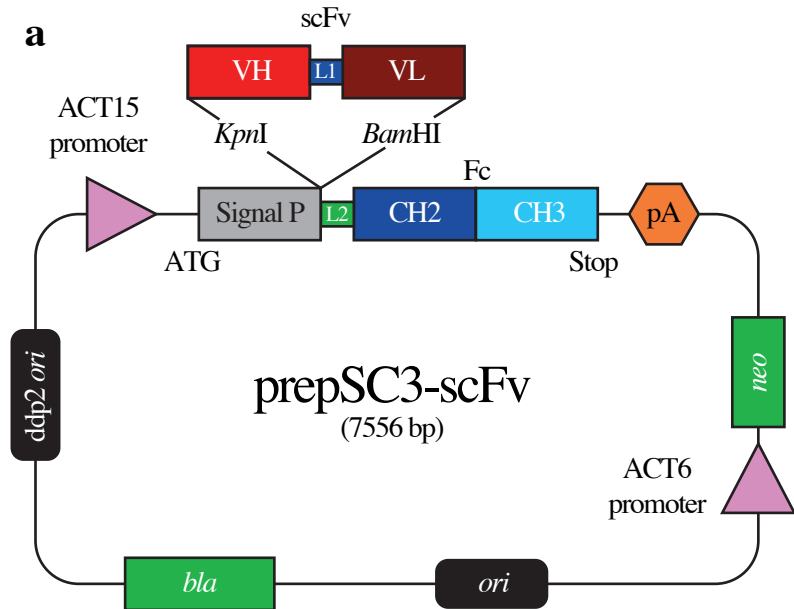**b**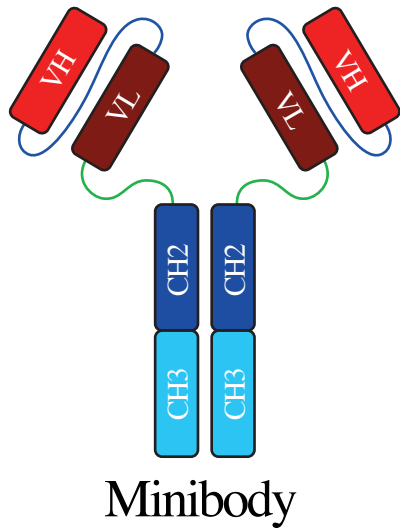

Supplement: Supplementary file 1 — Supplementary Material 1. Supplementary Fig. 1: Design of the expression vector prepSC3-scFv to produce recombinant antibodies in D. discoideum cells. a Schematic overview of the prepSC3 plasmid with the scFv fragment cloned in KpnI/BamHI. b Schematic of a minibody composed of a dimer of scFv-Fc fragments. (Signal P: signal peptide encoding sequence; VH and VL: variable domains of the heavy (VH) and light (VL) immunoglobulin chains; scFv: single-chain fragment variable; L1 and L2: peptide linkers; pA: D. discoideum polyadenylation and termination signal; neo: neomycin selection marker; ori: E. coli origin of replication; bla: beta-lactamase encoding gene. [file 13104_2025_7314_MOESM1_ESM.pdf]
